# Dual-Effect of S-Scheme Heterojunction and CQDs Strengthens the Charge Separation and Transfer in CQDs-g-C_3_N_4_/TiO_2_ Photocatalysts Toward Efficient Tetracycline Degradation

**DOI:** 10.3390/nano16030181

**Published:** 2026-01-28

**Authors:** Kunping Wang, Xiaojiang Su, Zhangxi Zhou, Liangqing Hu, Hao Li, Junyi Long, Ying Feng, Xiaobo Zhang, Jinghuai Zhang, Jing Feng

**Affiliations:** 1 Key Laboratory of Superlight Materials & Surface Technology, Ministry of Education, Harbin Engineering University, Harbin 150001, China; 2 College of Life Sciences, Tonghua Normal University, Tonghua 134000, China; 3 Jiangsu Key Laboratory of Advanced Structural Materials and Application Technology, Nanjing Institute of Technology, Nanjing 211167, China

**Keywords:** S-scheme heterojunction, carbon quantum dots, g-C_3_N_4_, TiO_2_, photocatalysis, tetracycline

## Abstract

Photocatalytic degradation of tetracycline (TC) is considered a viable technology due to its stable molecular structure and resistance to absorption by biological organisms. As a promising photocatalyst, TiO_2_ suffers from a wide bandgap and rapid charge recombination rates. In this work, the S-scheme heterojunctions of g-C_3_N_4_/TiO_2_ (CNTOx, x = 10, 30, and 70) were synthesized via solvothermal, calcination, and impregnation methods. Furthermore, carbon quantum dots (CQDs) were incorporated into the CNTO30 samples, resulting in yCQDs-CNTO30 (y = 0.5, 1, and 3). The 1CQDs-CNTO30 demonstrat an impressive TC degradation efficiency of 76.7% in 60 min under visible light, which is higher than that of CNTO30 (59.8%). This enhanced efficiency is ascribed to the effective charge separation induced by the dual-effect of S-scheme heterojunction and the CQDs. The built-in electric field within the heterojunction drives the separation of electrons and holes. Meanwhile, the highly conductive CQDs accelerate the electron transport, thereby promoting the charge separation. Additionally, the CQDs improve the ability of absorption light. This research provides critical insights into the strategic development of efficient ternary photocatalytic S-scheme heterojunctions for environmental remediation.

## 1. Introduction

Antibiotics are one of the most widely utilized drugs in both human and veterinary practices [1]. Consequently, the severity of antibiotic pollution is receiving increasing attention due to the rising global consumption and environmental discharge of these substances [2,3]. Among the common antibiotics, tetracycline (TC) is one of the most frequently used and widely detected in the natural water bodies, contributing to the proliferation of antibiotic resistance genes within bacterial populations [4,5,6]. The most remarkable environmental risk posed by antibiotics is the development of antibiotic resistance [7]. The aquatic ecotoxicity of TC primarily involves growth inhibition of photosynthesis and chlorophyll synthesis in the cyanobacteria and green algae.

In addition, the TC promotes the proliferation of antibiotic resistance genes and enriches resistant bacteria, which are disseminated via hydrological cycles and ecological networks, presenting a long-term direct threat to ecosystem stability and human health [1,3]. The antibiotics primarily enter into the surface water environment through the discharge of wastewater treatment plant effluents, direct release of wastewater from specific industries, agricultural and urban surface runoff. The dispersion pathways of TC in the surface water include urban sewage discharge (the primary pathway), directly discharge of medical wastewater, agricultural and aquaculture runoff, as well as atmospheric deposition and inter-basin transport [2]. Their spatial distribution and concentration levels are collectively influenced by multiple factors, including geography, hydrology, meteorology, and the intensity of regional human activities [1,8,9]. However, the TC exhibits persistent environmental behavior due to its inherent chemical stability and resistance to complete biodegradation by microbial communities [10,11]. Thus, its removal is crucial for mitigating potential risks to ecosystems and human health.

Degradation of TC by photocatalysts is considered a green redox technology, which generates redox active species through solar light excitation to induce oxidative degradation reactions [6,12]. The essential research for improving photocatalytic performance primarily focuses on enhancing light absorption, electron (e^−^)-hole (h^+^) separation efficiency, as well as the oxidation or reduction properties of h^+^ or e^−^. The TiO_2_ is regarded as one of the most promising photocatalysts due to its high stability, environmental friendliness, low cost, and excellent redox capabilities, which make it suitable for various applications such as water purification, air remediation, and self-cleaning surfaces [13,14]. Nevertheless, the practical implementation of bare TiO_2_ is mainly limited by its wide bandgap (~3.2 eV) and the rapid recombination of photogenerated electron–hole pairs [15]. Various modification strategies have been developed, including the deposition of a single atom (e.g., Pt, Au, carbon dots) [16] and the construction of heterojunctions [17]. These approaches extend the optical response of TiO_2_ into the visible range and enhance charge separation efficiency, thereby improving its photocatalytic performance under solar illumination [12,18].

The S-scheme heterojunction meets these requirements and exhibits the S-type electron migration driven by the built-in electric field (IEF) [18,19]. Generally, the S-scheme heterojunction is composed of two bad gap matched semiconductors [18,20,21]. The built-in electric field is formed owing to the different positions of the Fermi level (E_f_) at the interface. The IEF effectively separates carriers generated by light and maintains the robust redox properties of e^-^ and h^+^ [19]. The previous reports indicate that TiO_2_-based S-scheme heterojunctions, such as TiO_2_/COF and BiOBr/TiO_2_, display high photocatalytic efficiency, due to their ability to overcome the drawbacks associated with a wide bandgap and a high e^−^-h^+^ recombination rate [22,23,24,25]. For example, Moon et al. found that the Pt/g-C_3_N_4_/TiO_2_/IrOx (PCTI) hollow sphere photocatalyst demonstrated a photocatalytic H_2_ evolution rate of 8.15 mmol h^−1^ g^−1^. Its performance was attributed to the Z-scheme heterojunction optimized light absorption, charge separation, and reaction kinetics [26]. Li et al. synthesized the SubPc-5/TiO_2_ heterojunction with photocatalytic degradation of oxytetracycline (90%) and TC (99%), ascribing to the efficient photogenerated charge separation of the heterojunction [27]. Therefore, the S-scheme heterojunction is regarded as a highly effective approach for promoting the photocatalytic efficiency of TiO_2_ [23,28].

The narrow bandgap of g-C_3_N_4_ (2.58 eV) facilitates the absorption of visible light and the separation of photogenerated carriers within the S-scheme heterojunction [29,30,31,32,33,34]. In a S-scheme heterojunction, the positions of conduction band (CB) and valence band (VB) of g-C_3_N_4_ are both higher than those of TiO_2_. Consequently, g-C_3_N_4_ acts as a reductive semiconductor, enabling effective band alignment with the oxidative semiconductor TiO_2_ [35,36,37,38,39]. After close contact, the difference in work function between TiO_2_ and g-C_3_N_4_ drives electrons to transfer from g-C_3_N_4_ to TiO_2_, resulting in an IEF. This effectively separates charge carriers and remains a strong redox capability. For instance, Jiang et al. found that the photocatalytic water splitting activity of TiO_2_ nanodots/g-C_3_N_4_ was superior to that of TiO_2_ nanodots (by a factor of 2.2) and g-C_3_N_4_ (by a factor of 1.7) [40]. Furthermore, Jiang et al. reported that the S-scheme heterojunction photocatalyst (3DOM g-C_3_N_4_/TiO_2_) exhibited a higher photocatalytic activity for the H_2_O_2_ production [41]. This enhancement was attributed to the efficient separation of photogenerated charge carriers along with distinct reduction and oxidation sites in the S-scheme heterojunction [40,41].

Nevertheless, the high electron transport efficiency further enhances the separation of electrons and holes in an S-scheme heterojunction [42]. Typically, co-catalysts, such as Pt, Au, Cu, and carbon-based materials, which display high electrical conductivity, are employed [43,44,45]. The advantages of carbon quantum dots (CQDs), such as non-metals, low cost, environmental friendliness, and high light absorption efficiency, make them excellent co-catalysts [46,47]. Therefore, the CQDs with excellent electrical conductivity are chosen to be loaded on the S-scheme heterojunction to improve both the capacity of light absorption and charge transfer [48,49]. The combination of CQDs with a heterojunction provides more charge transfer active sites, serving as atomic-scale electron bridges, which facilitate carrier separation and drive rapid electron migration [42,48]. Meanwhile, the black color of CQDs broadens the light absorption capacity, thereby synergistically enhancing the overall photocatalytic performance [50,51]. For example, Che et al. found degradation efficiency of nitrogen-doped CQDs-modified Bi_2_WO_6_/g-C_3_N_4_ is 1.2 times that of the Bi_2_WO_6_/g-C_3_N_4_ photocatalyst [52]. This indicates that the composite containing the CQDs significantly enhances the photocatalytic performance. Shi et al. discovered that the CQDs, acting as electron acceptors, promote electron transfer. This enhanced the hydrogen production efficiency of the ZnInS_4_/quantum dot/g-C_3_N_4_ heterojunction by nearly tenfold [53].

Enhancing the separation of photogenerated carriers is a crucial strategy for improving photocatalytic efficiency in the photocatalytic process. Herein, the built-in electric field in the S-scheme heterojunction of g-C_3_N_4_/TiO_2_ facilitates the separation of charge carriers. Moreover, the incorporation of CQDs with the excellent electrical conductivity further enhances the electron migration ability and promotes the carrier separation. The CQDs also play a role in enhancing light absorption. Consequently, the ternary photocatalysts composited with S-scheme heterojunction g-C_3_N_4_/TiO_2_ and CQDs were synthesized. The influence of the compositional ratio among the g-C_3_N_4_, TiO_2_, and CQDs on the degradation of TC was systematically investigated. Furthermore, the roles of built-in electric field and CQDs in enhancing photocatalytic performance were also thoroughly discussed. The mechanism of photocatalytic degradation of TC in the reaction system was studied by quenching experiments and other exploratory experiments about the mechanism. It was found that the CQDs/g-C_3_N_4_/TiO_2_ S-scheme heterojunction photocatalyst displays excellent ability of charge transfer and visible light absorption, thereby boosting the photocatalytic performance of the catalyst. This research provides insights into improving the rapid transfer of electrons and the separation of e^−^-h^+^ pairs in the S-scheme heterojunctions.

## 2. Materials and Methods

### 2.1. Materials

All chemical reagents used in this work were of analytical grade and used without further purification. They were Tetrabutyl titanate (C_16_H_36_O_4_Ti, Tianjin Ruitejin Chemicals Co., Ltd., Tianjin, China), acetic acid (C_2_H_4_O_2_, Tianjin Fuyu Fine Chemical Co., Ltd., Tianjin, China), melamine (C_3_H_6_N_6_, Tianjin Guangfu Fine Chemical Research Institute, Tianjin, China), and citric acid (C_6_H_8_O_7_, Sinopharm Group Chemical Reagent Co., Ltd., Shanghai, China).

### 2.2. The Synthesis of TiO_2_

TiO_2_ was synthesized by a solvothermal method [54]. Tetrabutyl titanate (2 mL) and acetic acid (60 mL) were uniformly stirred for 10 min. Subsequently, the mixture was then heated under the solvothermal conditions at 140 °C for 12 h. The resulting powder was washed alternately with deionized water and anhydrous ethanol, and then dried to obtain the final sample (labeled as TiO_2_).

### 2.3. The Synthesis of g-C_3_N_4_

The g-C_3_N_4_ was synthesized by a thermal polymerization method, according to the previous work [31]. The synthesis process involved heating 10 g of melamine at 550 °C for 4 h in a muffle furnace, followed by cooling to 25 °C. The obtained bulk g-C_3_N_4_ was fully ground and calcined in the air at 500 °C for 2 h (heating rate: 5 °C min^−1^) again. Subsequently, it was quickly immersed in the liquid nitrogen (500 mL) for exfoliation, thus the g-C_3_N_4_ nanosheets were collected.

### 2.4. The Synthesis of g-C_3_N_4_/TiO_2_ S-Scheme Heterojunction

The g-C_3_N_4_/TiO_2_ composite was fabricated by an ultrasonic-calcination method [55]. A certain amount of the above obtained g-C_3_N_4_ nanosheets (20, 60, and 140 mg) was sonicated with 60 mL of anhydrous ethanol to obtain a uniform suspension for 2 h. Then, the as-synthesized TiO_2_ (0.2 g) was added to the suspension. The mixture was stirred for 12 h and then dried at 60 °C for 24 h. Finally, it was heated in a muffle furnace at 500 °C for 2 h with a heating rate of 5 °C min^−1^. The obtained g-C_3_N_4_/TiO_2_ composites were labeled as CNTO10, CNTO30, and CNTO70, respectively, corresponding to the different mass ratios of g-C_3_N_4_ to TiO_2_.

### 2.5. The Synthesis of CQDs

The CQDs were synthesized by a solvothermal method. A mixture of citric acid (5.88 g) and ethylenediamine (1876 µL) was dissolved in 70 mL of deionized water. This solution was treated at 180 °C for 12 h in a 100 mL Teflon-lined autoclave. The obtained brownish-black transparent solution was dialyzed using a dialysis bag (molecular weight cutoff: 3500, MD34-3500, Hunan Yibo Biotechnology Co., Ltd., Changsha, China) to yield the CQDs. Finally, the resulting solution was dried to obtain the CQDs.

### 2.6. The Preparation of yCQDs-g-C_3_N_4_/TiO_2_

**Figure 1:**
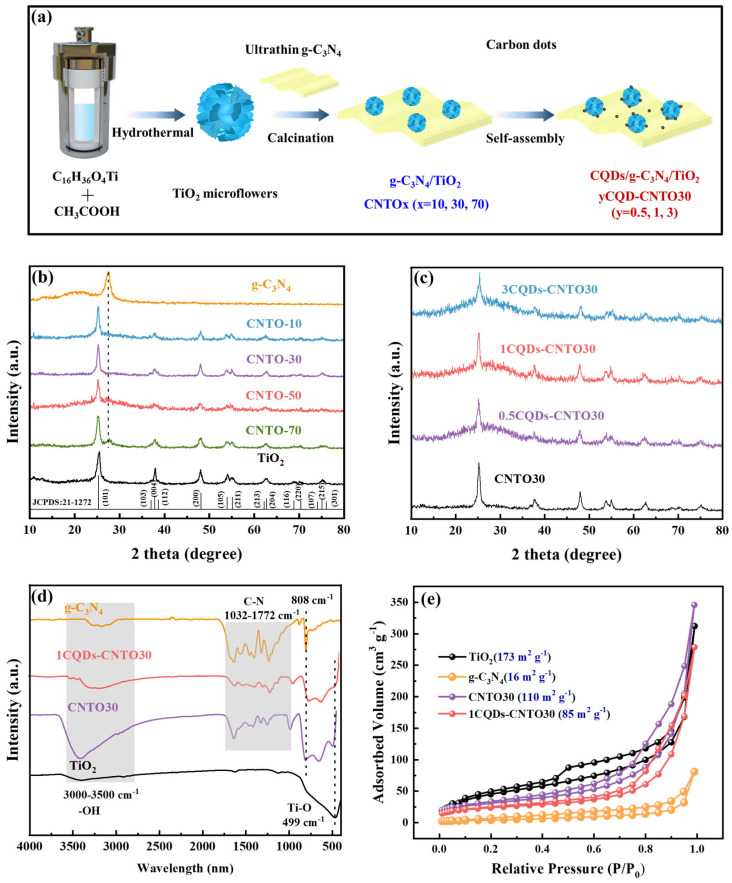
(**a**) The preparation route diagram of CQDs/g-C_3_N_4_/TiO_2_, (**b**) XRD patterns of TiO_2_, g-C_3_N_4_, and CNTOx (x = 10, 30, and 70), (**c**) yCQDs-CNTO30 (y = 0.5, 1, and 3), (**d**) FT-IR, and (**e**) nitrogen adsorption–desorption curves.

A 15 mL dispersion solution containing CQDs (0.0005, 0.001, or 0.003 g) and the CNTO30 (0.1 g) was prepared in accordance with the mass ratios of CQDs to g-C_3_N_4_/TiO_2_ heterojunction, which were 0.5%, 1%, and 3%, respectively. After stirring for 24 h, the resulting product was dried at 80 °C for 12 h. Finally, the samples containing 0.5%, 1%, and 3% mass fractions of CQDs were labeled as 0.5CQDs-CNTO30, 1CQDs-CNTO30, and 3CQDs-CNTO30, respectively (Figure 1a).

### 2.7. Characterization and Measurements

The details of characterization, photocatalysis measurements, and electrochemical measurements are in the Supporting Information.

## 3. Results and Discussion

### 3.1. Structure and Morphology

The X-ray diffraction (XRD) patterns reveal that the pure TiO_2_ pattern (Figure 1b) matches perfectly with the standard JCPDS card No. 21-1272, which corresponds to the anatase TiO_2_ [25]. In the case of pure g-C_3_N_4_, two broad diffraction peaks at 13.5° and 27.6° correspond to the (100) and (002) crystal planes, respectively. The (100) peak is assigned to the in-plane structural repeating units of 3 s triazine rings, while the (002) peak arises from the interlayer stacking of aromatic segments in g-C_3_N_4_ [56]. The diffraction peaks of both g-C_3_N_4_ and TiO_2_ are observed in the XRD patterns of g-C_3_N_4_/TiO_2_ (CNTOx, x = 10, 30, and 70). The intensity of diffraction peak at 27.6° progressively enhances with the increasing content, confirming the effective formation of g-C_3_N_4_/TiO_2_ composite structures. After the deposition of CQDs, a broad peak at approximately 23° is detected in the yCQDs-CNTO30 samples (y = 0.5, 1, and 3), suggesting the successful loading of CQDs onto the CNTO30 photocatalyst (Figure 1c). Moreover, the other diffraction peaks correspond well with those of CNTO30, demonstrating that the deposition of CQDs does not affect the crystal structure [57].

In Figure 1d, the Fourier-transform infrared spectroscopy (FT-IR) spectra for all samples are presented. The absorption peaks observed at about 3000–3500 cm^−1^ correspond to the stretching vibrations of the O-H bond in water molecules. Additionally, the absorption peak at 499 cm^−1^ is associated with the stretching vibration modes of the Ti-O bond and the Ti-O-Ti bond. For g-C_3_N_4_, the peak at 808 cm^−1^ is attributed to the stretching vibrations of the 3 s triazine ring structure. Meanwhile, the absorption bands located between 1032 cm^−1^ and 1772 cm^−1^ are assigned to the C-N stretching vibrations within the g-C_3_N_4_ framework. Notably, the characteristic absorption peaks of g-C_3_N_4_ are observed in both CNTO30 and 1CQDs-CNTO30, confirming the successful fabrication of the g-C_3_N_4_/TiO_2_ composite materials [58,59]. The specific surface area and pore structure properties of the catalysts were characterized by standard N_2_ adsorption–desorption measurements at 77 K. The corresponding isotherms for TiO_2_, g-C_3_N_4_, CNTO30, and 1CQDs-CNTO30 photocatalysts (Figure 1e) present typical type-IV isotherms with H3-type hysteresis loops, suggesting stacked mesoporous structures. The specific surface areas of TiO_2_, g-C_3_N_4_, CNTO30, and 1CQDs-CNTO30 photocatalysts are 173, 16, 110, and 85 m^2^·g^−1^, respectively. The observed decrease in specific surface area after the incorporation of g-C_3_N_4_ and CQDs is attributed to the inherently low surface area of g-C_3_N_4_ and pore blockage caused by the loading of CQDs. The pore size distribution curves confirm that all samples remain mesoporous characteristics, as shown in Figure S1.

**Figure 2:**
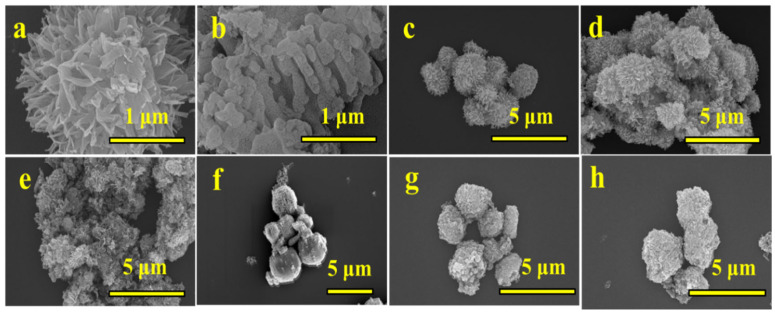
SEM images of (**a**) TiO_2_, (**b**) g-C_3_N_4_, (**c**) CNTO10, (**d**) CNTO30, (**e**) CNTO70, (**f**) 0.5CQDs-CNTO30, (**g**) 1CQDs-CNTO30, and (**h**) 3CQDs-CNTO30.

The morphologies of the obtained photocatalyst were characterized via scanning electron microscopy images (SEMs) and transmission electron microscopy (TEM). The morphologies of bare g-C_3_N_4_ and TiO_2_ are observed as thin sheets and flower-like structures, respectively (Figure 2a–e) [60]. The morphologies of the CNTO10, CNTO30, and CNTO70 demonstrate that g-C_3_N_4_ sheets are uniformly interspersed and grown on the sheets of TiO_2_ flower. As illustrated in Figure 2f–h, a higher content of CQDs in the 0.5/1/3CQDs-CNTO30 results in increased the roughness of surface.

**Figure 3:**
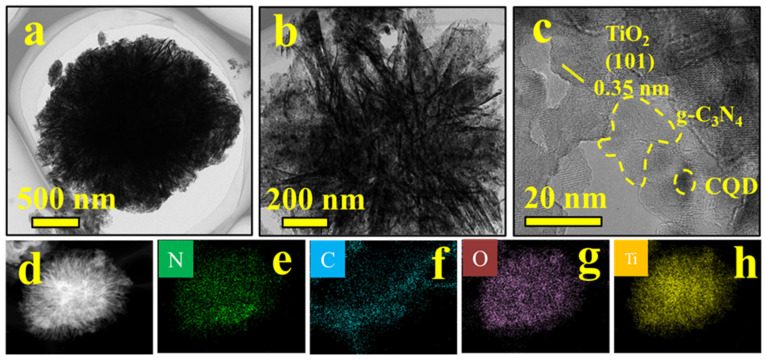
TEM images of 1CQDs-CNTO30, (**a**,**b**) TEM, (**c**) HRTEM, and (**d**–**h**) EDS images (**d**) STEM image, (**e**) N, (**f**) C, (**g**) O, (**h**) Ti.

In particular, the TEM images of 1CQDs-CNTO30 (Figure 3a,b) exhibit a composite shape of flower-like and sheet-like, which is consistent with the SEM images. The (101) crystal plane of TiO_2_ and the amorphous region of g-C_3_N_4_ are detected in the high-resolution transmission electron microscopy (HRTEM) image (Figure 3c), confirming the successful composite formation [61,62]. Notably, the average diameters of the CQDs were measured to be 11.4 nm (Figure S3). As a result, the HRTEM images indicate that the CQDs are well-dispersed without significant agglomeration, which is crucial for their effective role as discrete electron transfer mediators. Figure 3d–h illustrate the uniform distribution of Ti, O, C, and N elements in the Energy Dispersive X-ray Spectroscopy (EDS) of 1CQDs-CNTO30. This indicates that g-C_3_N_4_ and CQDs are successfully dispersed on the surface of TiO_2_.

### 3.2. The Valence States and Element Composition

**Figure 4:**
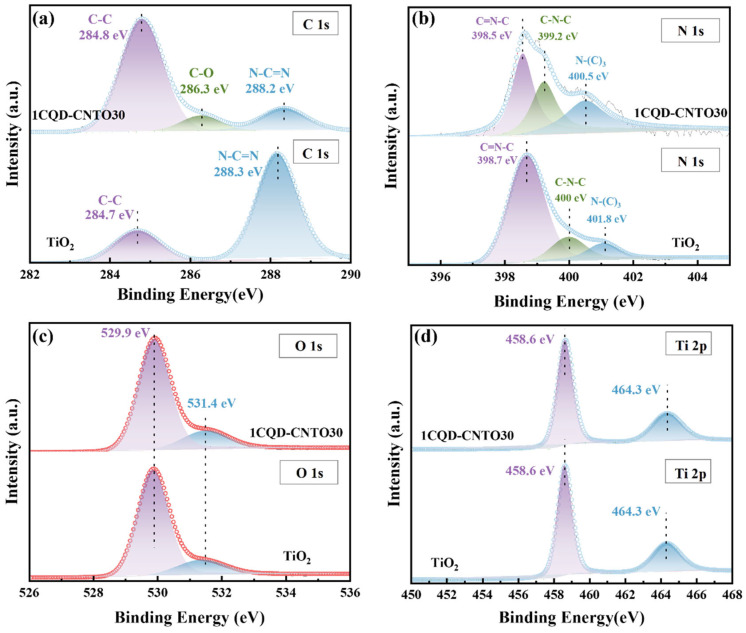
The XPS spectra of TiO_2_, g-C_3_N_4_, and 1CQDs-CNTO30, (**a**) C *1s*, (**b**) N *1s*, (**c**) O *1s*, (**d**) Ti *2p*.

To determine the elemental composition and oxidation states of 1CQDs-CNTO30, g-C_3_N_4_, and TiO_2_, the X-ray photoelectron spectroscopy (XPS) analysis was conducted. As illustrated in Figure 4a, the C 1s spectrum for 1CQDs-CNTO30 is decomposed into three distinct peaks at 288.2, 286.3, and 284.8 eV, which correspond to N-C=N bonds, C-O bonds, and C-C bonds, respectively [63]. It is important that the C-O bond is absent in g-C_3_N_4_ but present in 1CQDs-CNTO30, indicating a chemical interaction of Ti-O-C bonds connecting TiO_2_ and g-C_3_N_4_. The N *1s* spectrum of g-C_3_N_4_ exhibits three peaks at 401.8, 400.0, and 398.7 eV, attributed to N-(C)_3_, C-N-C, and C=N-C bonds (Figure 4b), respectively [64]. Notably, these nitrogen-related peaks of 1CQDs-CNTO30 shift toward lower binding energies, suggesting electron transfer from TiO_2_ to g-C_3_N_4_ and strong interfacial interaction between them [26,27,65]. As shown in Figure 4c, both TiO_2_ and 1CQDs-CNTO30 display O *1s* peaks at 529.9 and 531.4 eV, corresponding to lattice oxygen and surface hydroxyl groups, respectively. Their Ti *2p* spectra present two distinct peaks at 458.6 and 464.3 eV, assigned to Ti^4+^ *2p*_3/2_ and Ti^4+^ *2p*_1/2_ spin–orbit components (Figure 4d). Additionally, the XPS results provide the further evidence for the successful synthesis of composite samples [66].

### 3.3. The Photocatalytic Performance

#### 3.3.1. The Effect of g-C_3_N_4_ and CQDs Ratio on the Photoatalytic Performance

**Figure 5:**
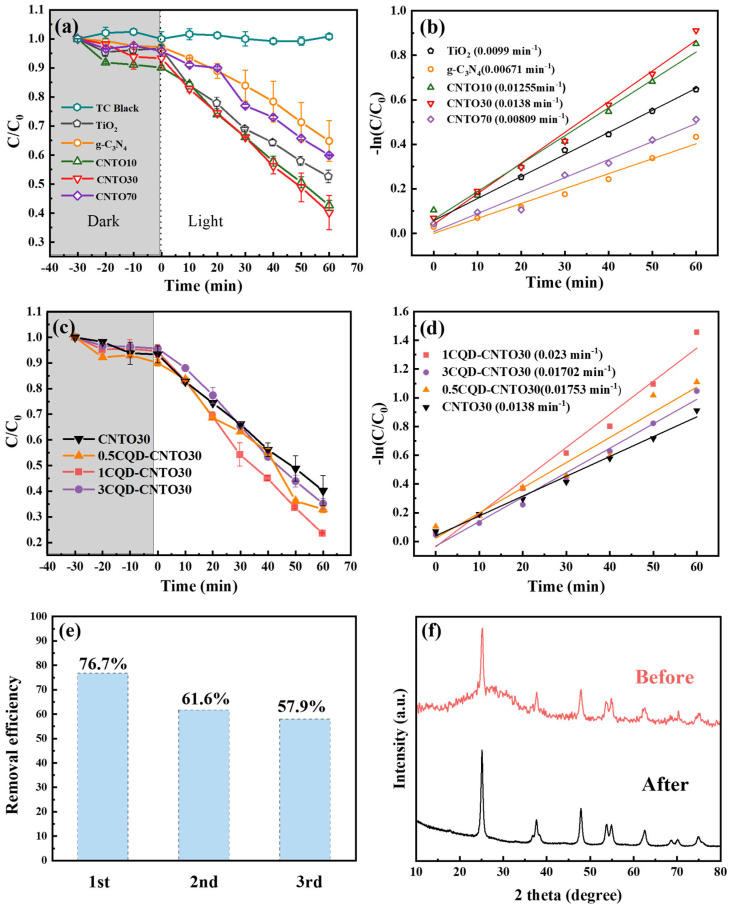
(**a**) The photocatalytic degradation efficiency of TC and (**b**) kinetic fitting of TiO_2_, g-C_3_N_4_, CNTOx (x = 10, 30, and 70), (**c**) photocatalytic degradation efficiency of TC and (**d**) kinetic fitting of yCQDs-CNTO30 (y = 0.5, 1, and 3), (**e**) the photocatalytic degradation for three cycles of 1CQDs-CNTO30, (**f**) XRD patterns of 1CQDs-CNTO30 before and after recycling tests.

Figure 5 presents the photocatalytic degradation efficiencies of TiO_2_, g-C_3_N_4_, CNTOx (x = 10, 30, and 70), and yCQDs-CNTO30 (y = 0.5, 1, and 3) catalysts. Initially, the photocatalysts underwent a 30 min adsorption experiment under dark condition until adsorption equilibrium was achieved. It is evident that the adsorption performance of all photocatalysts is low (the gray area in Figure 5). Then, the photocatalytic experiment was performed under simulated sunlight. Figure 5a shows that the degradation of individual TC under simulated sunlight is negligible.

The photocatalytic degradation efficiencies of TiO_2_, g-C_3_N_4_, CNTO10, CNTO30, and CNTO70 were 47.61%, 35.18%, 57.3%, 59.8%, and 40.62%, respectively. It can be found that the photocatalytic efficiency of CNTOx (x = 10, 30, and 70) composites initially increases and then decreases with higher content of g-C_3_N_4_. The optimal performance is a loading of 30 wt% g-C_3_N_4_ (CNTO30). However, excessive integration of g-C_3_N_4_ acts as a recombination center for photogenerated e^−^-h^+^ pairs and simultaneously reduces the specific surface area, thereby suppressing overall photocatalytic efficiency [36,67]. Figure 5b illustrates the kinetic fitting curves of these catalysts, where the reaction rate constants (*k*) exhibit a trend consistent with the degradation efficiencies. The CNTO30 exhibits the highest *k* value (0.0014 min^−1^), which is 1.4 times and 2.1 times higher than those of bare TiO_2_ and g- g-C_3_N_4_, respectively. These findings suggest that the incorporation of g-C_3_N_4_ at a moderate level improves the photocatalytic activity of TiO_2_.

According to the above results, the optimal loading ratio of g-C_3_N_4_ is 30 wt% (CNTO30) for the g-C_3_N_4_/TiO_2_ composite photocatalyst. Next, the CQDs are deposited onto CNTO30. As depicted in Figure 5c,d, the TC photocatalytic degradation efficiencies of 0.5CQDs-CNTO30, 1CQDs-CNTO30, and 3CQDs-CNTO30 are 67.0%, 76.7%, and 64.9%, respectively. These values are higher than that of the bare CNTO30, which achieves only 59.8% degradation under the identical conditions. This result confirms that the incorporation of CQDs effectively promotes photocatalytic performance. Importantly, the 1 wt% CQDs loading achieves the highest TC degradation efficiency (76.7%) and the fastest reaction rate constant (*k* = 0.023 min^−1^). However, the increase in CQD loading to 3 wt% results in a decrease in photocatalytic efficiency. The excessive concentration of CQDs disrupts the ordered recombination of electrons. This disordered recombination process significantly reduces the carrier utilization efficiency [68,69]. Table 1 shows the comparison of the degradation rate for TC using different photocatalysts [70,71,72,73,74,75,76,77]. The degradation rate (υ) of the photocatalyst can be defined as

(1) $$\upsilon =\frac{1}{m_{\mathrm{cat}}}\;\times \;\frac{E\%\;\times {\;m}_{\mathrm{TC}}}{t}$$

where *m_cat_* is the mass of catalyst (g_cat_), *m_TC_* is the mass of TC (g), *E*% is the photocatalytic degradation removal efficiency, and *t* is photocatalytic time (min) [78]. The calculated value is defined as the 1 g of TC degraded by a 1 g of catalyst per minute. It is found that the degradation rate υ of 1CQDs-CNTO30 is 3.2 × 10^−3^ g g_cat_^−1^ min^−1^, which is relatively higher. This indicates that the synergistic effect of CQDs and S-scheme heterojunctions effectively enhances the separation of photogenerated electrons. To further validate the potential of 1CQDs-CNTO30 in practical applications, its degradation performance for trichlorobenzene was tested in the simulated drinking water (tap water) and actual water bodies (lake water). The obtained results presented that the degradation efficiencies of TC in tap and lake water were 48.2% and 41.4%, respectively, lower than that in deionized water. This reduction is ascribed to the presence of acidity, salinity, dissolved oxygen, microorganisms, metal ions, and turbidity in tap and lake water, which hinder the absorption of light and cause photogenerated reactive species compete with the target pollutant [79,80].

#### 3.3.2. The Cyclic Experiment

As illustrated in Figure 5e, the photocatalytic degradation efficiency of 1CQDs-CNTO30 decreased from 76.7% to 57.9% after three cycles. The XRD results of before and after the catalytic experiments reveal the disappearance of the broad peak at approximately 23° assigning to CQDs, as shown in Figure 5f which was attributed to the CQDs. This indicates that the CQDs detached from the catalyst matrix during the photocatalytic process, thereby accounting for the observed decline in photocatalytic performance.

#### 3.3.3. Optoelectronic Properties

**Figure 6:**
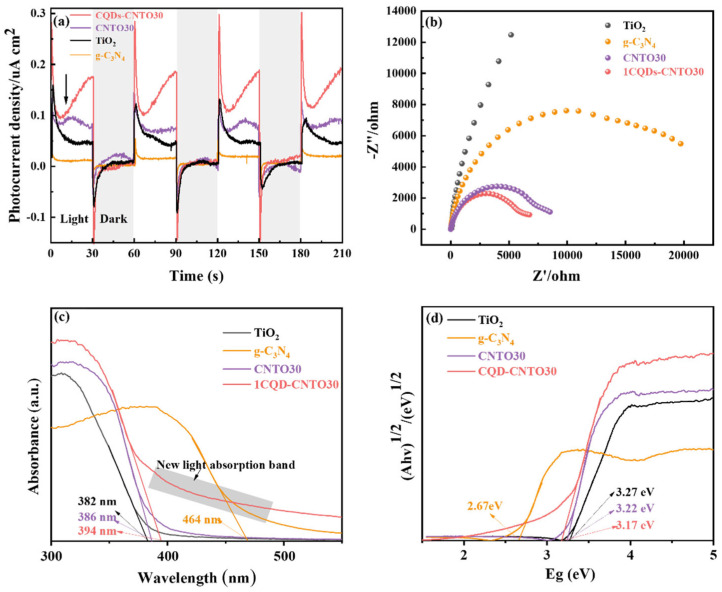
(**a**) The photoluminescence spectra, (**b**) electrochemical impedance spectra, (**c**) the UV–visible diffuse reflectance spectra, and (**d**) Tauc plots of TiO_2_, g-C_3_N_4_, CNTO30, and 1CQDs-CNTO30.

The photoelectrochemical properties were measured to elucidate the effect of heterojunction and CQD loading on the separation and migration of charge carriers. Figure 6a presents the curves of transient photocurrent response for the catalysts. The photocurrent density of CNTO30 is larger than that of both pure TiO_2_ and g-C_3_N_4_, suggesting that the heterojunction enhances the separation of charge carriers generated. Remarkably, the highest photocurrent density of 1CQDs-CNTO30 indicates that the incorporation of CQDs accelerates electron migration, which suppresses the recombination of charge carriers.

Moreover, electrochemical impedance spectroscopy (EIS) was employed to evaluate the surface electron transfer rates of the photocatalysts. In Figure 6b, the Nyquist semicircle radius of CNTO30 and 1CQDs-CNTO30 is smaller than that of TiO_2_ and g-C_3_N_4_, which suggests a reduced charge transfer resistance. These results demonstrate that the formation of heterojunction and incorporating CQDs effectively facilitates surface electron migration and transfer. Furthermore, these results agree with the diffuse reflectance spectroscopy (DRS). The UV-vis DRS spectra of TiO_2_, g-C_3_N_4_, CNTO30, and 1CQDs-CNTO30 photocatalysts were tested to detect the optical absorption properties. As revealed in Figure 6c, the absorption edges of TiO_2_, g-C_3_N_4_, CNTO30, and 1CQDs-CNTO30 are located at 382, 464, 386, and 394 nm, respectively. After loading of CQDs, a redshift in the absorption edge is observed, accompanied by the emergence of new absorption band in the visible region. This phenomenon is attributed to the up-conversion photoluminescence and the property of CQDs to reduce the reflection of light, indicating that CQDs enhance the light-harvesting capability [51,81]. The corresponding bandgap energies, calculated via the Tauc plot method (Figure 6d), are 3.25, 2.67, 3.22, and 3.09 eV for TiO_2_, g-C_3_N_4_, CNTO30, and 1CQDs-CNTO30, respectively meaning that the visible light responsiveness of TiO_2_ is enhanced by the incorporation of g-C_3_N_4_ and CQDs.

#### 3.3.4. The Band Structure Analysis

**Figure 7:**
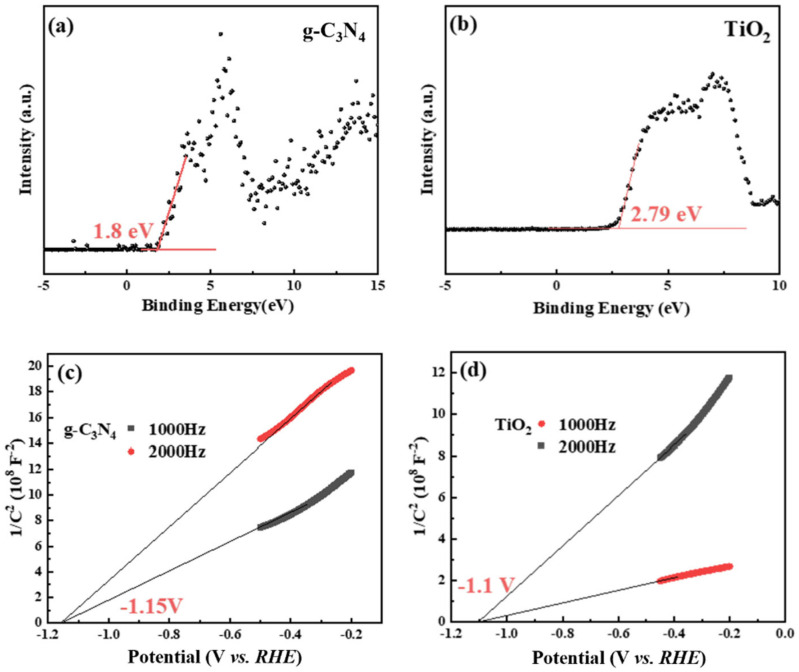
(**a**,**b**) The VB XPS spectra, and (**c**,**d**) the Mott–Schottky plots of TiO_2_ and g-C_3_N_4_.

The photocatalytic degradation process involves the migration of photogenerated charge carriers to the catalyst surface, initiating redox reactions that generate reactive oxygen species (ROS) to oxidize and degrade pollutants. The generation of ROS requires e^−^ or h^+^ to achieve the potential of −0.33 V (vs. *NHE*) for O_2_/O_2_^−^ or 2.27 V (vs. *NHE*) for H_2_O/·OH generation [82,83]. The band structure was analyzed and calculated via the DRS, valence band XPS (VB-XPS), and Mott–Schottky measurements. The VB-XPS spectra reveal that the energy differences between the valence band (VB) maximum and the Fermi level (E_f_) are 1.80 eV for g-C_3_N_4_ and 2.79 eV for TiO_2_ (Figure 7a,b). Concurrently, the flat-band potentials (E_FB_) of g-C_3_N_4_ and TiO_2_ are determined from Mott–Schottky curves, with E_FB_ approximating the E_f_ (Figure 7c,d). Consequently, the E_FB_ values for g-C_3_N_4_ and TiO_2_ are −1.15 V and −1.10 V (vs. *Ag*/*AgCl*, pH = 7), which convert to −0.54 V and −0.49 V (vs. *NHE*, pH = 7), respectively. Based on these values, the VB positions of g-C_3_N_4_ and TiO_2_ are calculated to be 1.26 eV and 2.30 eV, respectively. Utilizing the bandgap values derived from Tauc plots, the CB positions are determined to be −1.41 eV for g-C_3_N_4_ and −0.95 eV for TiO_2_. These values are listed in Table S1.

Based on the band structure analysis of g-C_3_N_4_ and TiO_2_, g-C_3_N_4_ exhibits a more negative E_f_ and CB position than TiO_2_ [36,37,40]. This unique electronic configuration enables g-C_3_N_4_ to form the S-scheme heterojunction with TiO_2_ to enhance the separation efficiency of photogenerated charge carriers.

#### 3.3.5. Reactive Species

**Figure 8:**
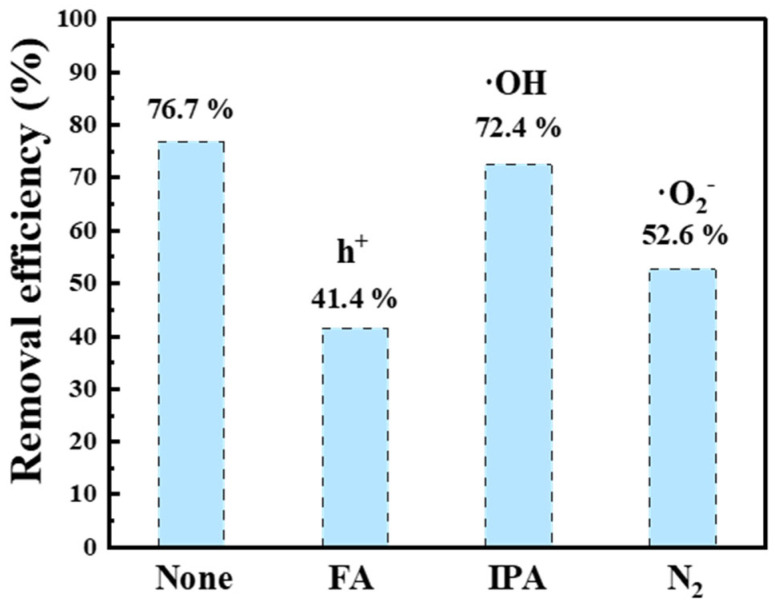
Quenching experiments of 1CQDs-CNTO30.

The quenching experiments were conducted to identify the reactive species that play a role in the degradation of TC within the system. The formic acid (FA) was utilized as a scavenger to capture holes (h^+^), isopropyl alcohol (IPA) served as a means to scavenge hydroxyl radicals (·OH), and nitrogen gas (N_2_) was employed to neutralize superoxide anions (·O_2_^−^) [24]. In Figure 8, the photocatalytic degradation efficiency to TC of 1CQDs-CNTO30 decreased from 76.7% to 41.4%, 72.4%, and 52.6% after the introduction of FA, IPA, and N_2_, respectively. These results indicate that h^+^ and ·O_2_^−^ are the primary reactive species responsible for TC degradation.

#### 3.3.6. The Mechanism of Photocatalytic Degradation

**Figure 9:**
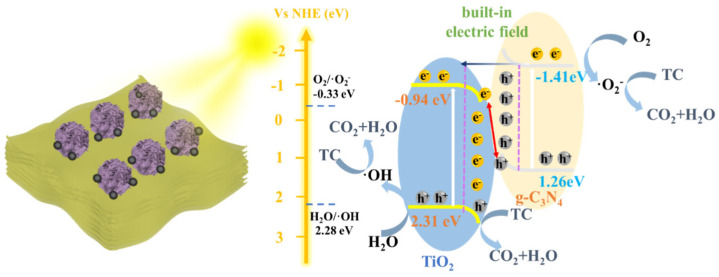
The mechanism diagram of photocatalytic degradation on the obtained catalyst.

The formation of S-scheme heterojunction and the incorporation of CQDs in the 1CQDs-CNTO30 promote the separation and migration of charge carriers while improving light absorption capabilities. As illustrated in Figure 9 and Equations (2)–(5), the UV-vis light excites the e^−^-h^+^ pairs. Driven by the built-in electric field, the h^+^ in VB-g-C_3_N_4_ recombines with the e^−^ in CB-TiO_2_ to achieve efficient carrier separation. This process maintains highly reactive e^−^ and h^+^ with strong redox capabilities for catalytic reactions. The h^+^ in VB-TiO_2_ directly oxidizes the TC. Simultaneously, the e^-^ in CB- g-C_3_N_4_ interact with dissolved oxygen in the water, generating superoxide radicals (·O_2_^−^). Additionally, the potential of the VB in TiO_2_ (2.3 eV (vs. *NHE*)) marginally surpasses the redox potential necessary for the production of (·OH) generation (2.27 eV (vs. *NHE*)), allowing the generation of ·OH radicals [24,52]. The detailed reaction mechanism is illustrated in Figure 9.

(2) $$1\mathrm{CQDs}\text{-}\mathrm{CNTO}30\to e^{-}+h^{+}$$

(3) $$O_{2}+e^{-}\to \cdot {O_{2}}^{-}$$

(4) $$H_{2}O+h^{+}\to \cdot \mathrm{OH}$$

(5) $$\mathrm{Org}.\;+\;h^{+},\cdot {O_{2}}^{-}\to {\mathrm{CO}}_{2}+H_{2}O$$

## 4. Conclusions

In conclusion, this study successfully fabricated a ternary carbon quantum dots (CQDs)/g-C_3_N_4_/TiO_2_ photocatalyst using solvothermal, calcination, and impregnation techniques for efficient degradation of tetracycline (TC) under visible light irradiation. The optimal composite material 1CQDs-CNTO30 (with 30 wt% g-C_3_N_4_ and 1 wt% CQDs) achieved a TC degradation efficiency of 76.7% in 60 min. This performance was significantly superior to those of TiO_2_ (47.61%) and CNTO30 (59.8%). The improved photocatalytic performance is attributed to the combined effects of the S-scheme heterojunction and the modification with the CQDs. The S-scheme heterojunction between g-C_3_N_4_ and TiO_2_ facilitates efficient charge separation by recombining holes (h^+^) from the valence band of g-C_3_N_4_ with electrons (e^−^) from the conduction band (CB) of TiO_2_, preserving high-redox-potential charge carriers. Meanwhile, the incorporation of CQDs expands the visible light absorption range through their up-conversion luminescence and electron transport properties, accelerates electron migration, and suppresses carrier recombination. The quenching experiments identified superoxide radicals (·O_2_^−^) and h^+^ as the major active species, hydroxyl radicals (·OH) serving as secondary contributors. It is found that the electrons from the CB of g-C_3_N_4_ react with dissolved oxygen to generate ·O_2_^−^, while h^+^ in VB-TiO_2_ directly oxidize TC. This study clearly demonstrates that the targeted construction of S-scheme heterojunctions combined with the CQDs constitutes an effective strategy for the efficient preparation of advanced photocatalysts. The findings suggest that such innovative photocatalytic systems not only exhibit superior performance but also hold substantial promise for practical applications in environmental remediation, addressing critical challenges in pollution treatment and environmental cleanup. The characteristics of CQDs/g-C_3_N_4_/TiO_2_ exhibit a great potential in applications, such as photocatalytic CO_2_ reduction, photocatalytic water splitting for H_2_ production, photocatalytic synthesis of H_2_O_2_, and green organic synthesis. These applications significantly contribute to global sustainability objectives, advancing the renewable energy adoption and carbon neutrality targets.

## Figures and Tables

**Table 1:** Comparison the performance of TC degraded with difference photocatalysts.

Reference	Photocatalyst	Degradation Removal Efficiency to TC (%) and k (min^−1^)	Degradation Conditions	Degradation Rate (υ) *
This work	1CQDs-CNTO30	76.7% 0.0023	C_TC_: 50 mg L^−1^; time: 60 min; m_cat_: 0.2 g L^−1^;	3.2 × 10^−3^
[70]	TiO_2_/g-C_3_N_4_	79.11% 0.01587	C_TC_: 100 mg L^−1^; time: 100 min; m_ca_t: 2 g L^−1^;	3.96 × 10^−4^
[71]	g-C_3_N_4_ NRs/TiO_2_	71% 0.0204	C_TC_: 20 mg L^−1^; time: 60 min; m_cat_: 0.1 g L^−1^	2.37 × 10^−3^
[72]	Membrane of g-C_3_N_4_/TiO_2_	66.1%	C_TC_: 50 μg L^−1^; time: 150 min; m_cat_: 0.03 wt%	7.34 × 10^−6^
[73]	GQDs/g-C_3_N_4_	80% 0.00128	C_TC_: 15 mg L^−1^; time: 120 min; m_cat_: 1 g L^−1^	1.00 × 10^−5^
[74]	g-C_3_N_4_ nanosheets	83% 0.013	C_TC_: 10 mg L^−1^; time: 120 min; m_cat_: 1 g L^−1^	6.92 × 10^−5^
[75]	N-rich g-C_3_N_4_	98% 0.0394	C_TC_: 30 mg L^−1^; time: 90 min; m_cat_: 0.5 g L^−1^	6.53 × 10^−4^
[76]	g-C_3_N_4_	86% 0.0024	C_TC_: 20 mg L^−1^; time: 360 min; m_cat_: 1 g L^−1^	4.78 × 10^−5^
[77]	Bi_2_W_2_O_9_/g-C_3_N_4_	95% 0.04284	C_TC_: 10 mg L^−1^; time: 90 min; m_cat_: 1 g L^−1^	1.06 × 10^−4^

* The photocatalytic activity was evaluated in terms of degradation rate per mass of catalyst, calculated by the $\upsilon \;=\;\frac{1}{m_{\mathrm{cat}}}\;\times \;\frac{E\%\;\times {\;m}_{TC}}{t}$. Where *m_cat_* is the mass of catalyst (g_cat_), *m_TC_* is the mass of TC (g), *E*% is the photocatalytic degradation removal efficiency, and *t* is photocatalytic time (min).

## Data Availability

Data are contained within the article and Supplementary Materials.

## Supplementary Materials

The following supporting information can be downloaded at: https://www.mdpi.com/article/10.3390/nano16030181/s1, Figure S1: Pore size distribution diagram of TiO_2_, g-C_3_N_4_, CNTO30, and 1CQDs-CNTO30; Figure S2: The survey of XPS of TiO_2_ and 1CQDS-CNTO30; Figure S3: Size distribution diagram of CQDs in 1CQDs-CNTO30; Figure S4: Actual water quality (tap and lake water) on TC degradation in the photocatalytic degradation of 1CQDs-CNTO30; Table S1: Energy level parameters: band gap, the position of CB values VB, and flat-band of different catalysts; Figure S5: (a) Surface charge variation as a function of the pH of TiO_2_, g-C_3_N_4_, CNTO30, and 1CQDs-CNTO30, and (b) The adsorption removal efficiency in 30 min. Reference [84] is cited in the Supplementary Materials.
