# Supplementary material for: Dual-Effect of S-Scheme Heterojunction and CQDs Strengthens the Charge Separation and Transfer in CQDs-g-C3N4/TiO2 Photocatalysts Toward Efficient Tetracycline Degradation
Source: Nanomaterials (Basel). 2026 Jan 28;16(3):181. doi: 10.3390/nano16030181 (PMC12899698; doi:10.3390/nano16030181)
Supplement: Supplementary file 1 [file nanomaterials-16-00181-s001.zip › nanomaterials-4097746-supplementary.pdf]

Dual-effect of S-scheme Heterojunction and CQDs Strengthens the Charge Separation and Transfer in CQDs-g-C_3N_4/TiO_2 Photocatalysts toward Efficient Tetracycline Degradation

Kunping Wang[a1], XiaoJiang Su[a1], Zhangxi Zhou[a], Liangqing Hu[a], Hao Li[a], Junyi, Long[a], Ying Feng[b], Xiaobo Zhang[c], JingHuai Zhang[a], Jing Feng[a*]

a Key Laboratory of Superlight Materials & Surface Technology of Ministry of Education, Harbin Engineering University, Harbin 150001, PR China

b College of Life Sciences, Tonghua Normal University, Tonghua 134000, China

c Jiangsu Key Laboratory of Advanced Structural Materials and Application Technology, Nanjing Institute of Technology

[*] Corresponding author. E-mail address: fengjing@hrbeu.edu.cn (Jing Feng)

1 These authors contributed equally.

1. Characterization

The crystal structure was analyzed by X-ray diffraction (XRD) using a Rigaku/TTR-III instrument with Cu-Kα radiation (wavelength λ = 0.15405 nm). The 2θ range was from 10 °~ 80 °. The surface properties of the samples were characterized by X-ray photoelectron spectroscopy (XPS) on a K-Alpha instrument (XSAM 800, Kratos, UK) with Al K-α (1361 eV) excitation source. Morphology was examined using scanning electron microscopy (SEM, HITACHI/S-4800) and transmission electron microscopy (TEM, FEI Tecnai G2 S-Twin, 200 kV). The band properties of the samples were evaluated by ultraviolet-visible (UV-vis) diffuse reflectance spectroscopy (UV-2450). Radical species were detected by electron spin resonance (ESR, BRUKER A300) using DMPO as the spin-trapping reagent.

2. Photocatalytic measurements

The detailed process for photocatalytic degradation is as follows. Simulated solar light was provided by a 300 W xenon lamp with a wavelength greater than 420 nm. The reactor was placed directly below the light source at a distance of 15 cm. 0.02 g of the as-prepared composite catalyst was added into the reactor, which contained TC wastewater (100 mL, 50 mg L^{-1}). First, adsorption was carried out in the dark for 20 min to achieve adsorption-desorption equilibrium. Subsequently, the photocatalytic reaction was initiated under the xenon lamp irradiation. At every 10 min interval, a certain amount of suspension was sampled. It was filtered through a microporous filter (0.22 μm). Quantitative analysis was performed during the illumination process. The

residual TC concentration was measured by spectrophotometry at 356 nm. The photocatalytic degradation efficiency was calculated using the following formula (1).

$$\eta = \frac{C_0 - C_t}{C_0} \quad (1)$$

Here, η is the degradation efficiency, C_0 is the initial TC concentration before irradiation, and C_t is the TC concentration at irradiation time t.

3. Electrochemical measurements

The electrochemical properties of the samples were measured using a three-electrode method on an electrochemical workstation (CHI 660E). The electrolyte was Na_2SO_4 (0.5 mol L^{-1}), a Pt electrode served as the counter electrode, and an Ag/AgCl electrode was used as the reference electrode. The slurry was a mixture of catalyst (5 mg), Nafion (20 μL), and isopropanol (1 mL). The slurry was ultrasonicated for 30 min and then coated onto an ITO glass. The coated electrode was dried under vacuum at 60 °C for 12 h to obtain the working electrode.

The Mott – Schottky plots were obtained using the Impedance-Potential mode. Measurements were performed at a fixed frequency (typically 1000 Hz) under dark conditions. Electrochemical Impedance Spectroscopy (EIS) were recorded using a Solartron analytical system in the frequency range from 0.01 Hz to 100 kHz under open-circuit potential conditions. The transient photocurrent response was measured in the chronoamperometry mode. The working electrode was illuminated intermittently (light on/off cycles of 30 seconds each), and the difference in current density under illuminated and dark conditions was used to evaluate the photocurrent generation capability of the catalyst.

4. The point of zero charge measurements

The point of zero charge (PZC) of the sample surfaces was determined using the Zeta potential method [1]. The catalyst was dispersed at a concentration of 0.01 g L^{-1} in a 1 mmol NaCl solution. Solutions with pH values of 3, 5, 7, 9, and 11 were prepared using HCl and NaOH solutions. The suspensions were allowed to stand for a sufficient period (36 h) to reach adsorption equilibrium. The Zeta potential of the samples at each pH was then measured. A curve was plotted with pH on the horizontal axis and Zeta potential on the vertical axis. The pH at which the curve intersected the origin (Zeta potential = 0) corresponds to the point of zero charge (pH_{PZC}).

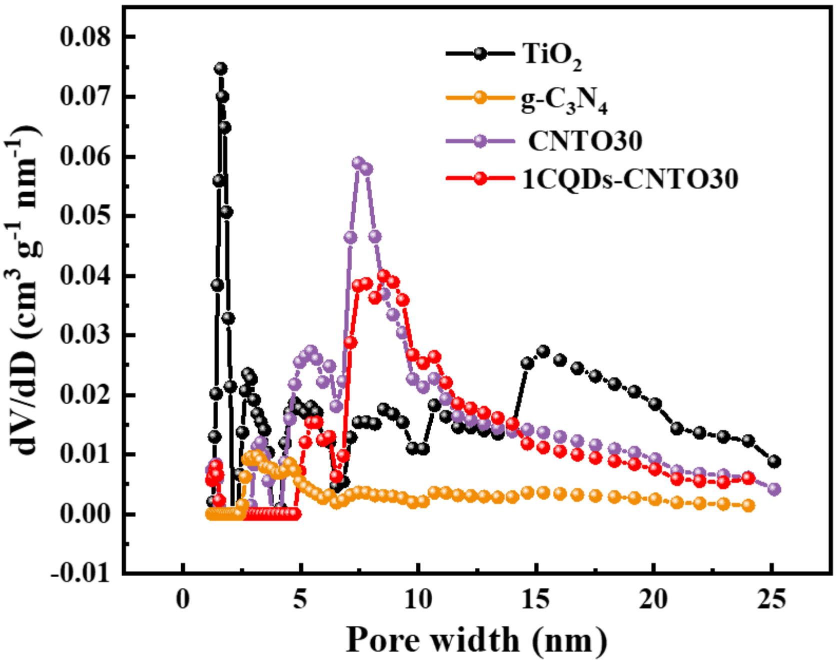

Fig. S1 Pore size distribution diagram of TiO_2, $g\text{-}C_3N_4$, CNTO30, and 1CQDs-CNTO30.

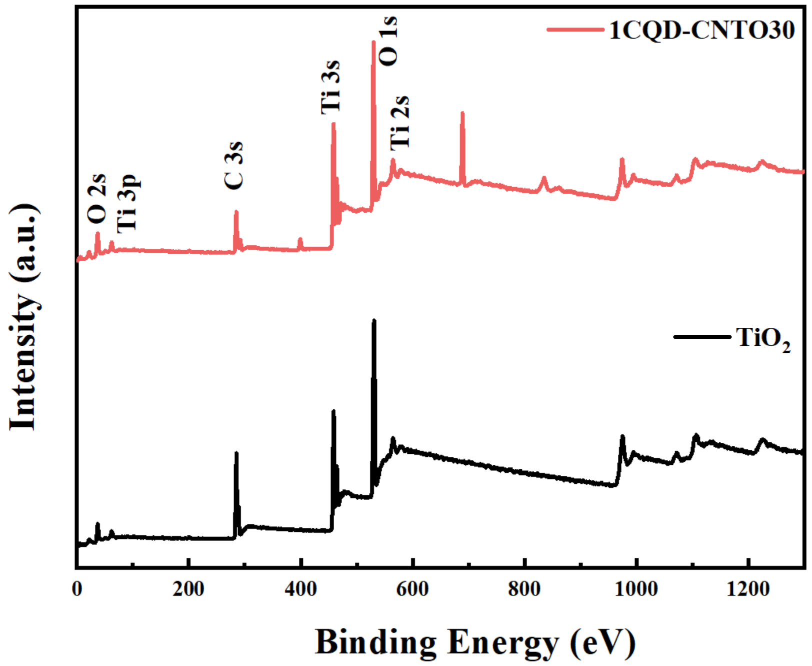

Fig. S2 The survey of XPS of TiO2 and 1CQDS-CNTO30.

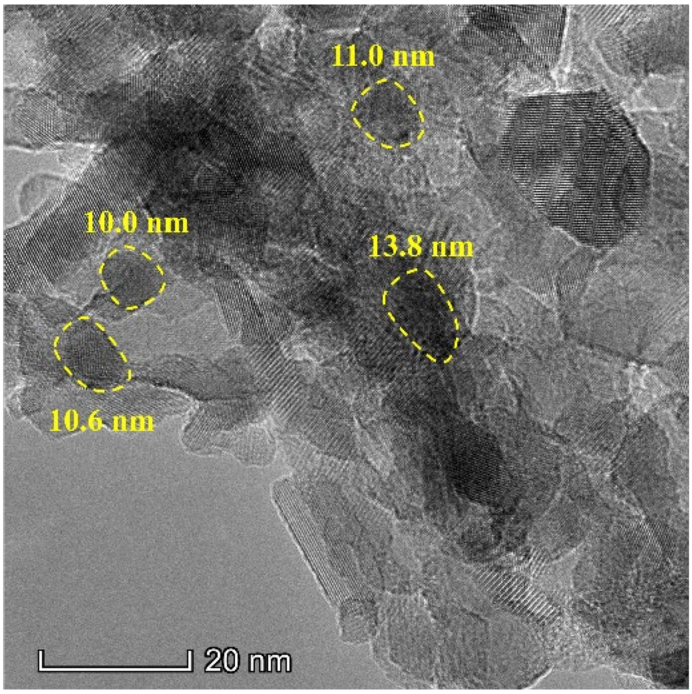

Fig. S3 Size distribution diagram of CQDs in 1CQDs-CNTO30

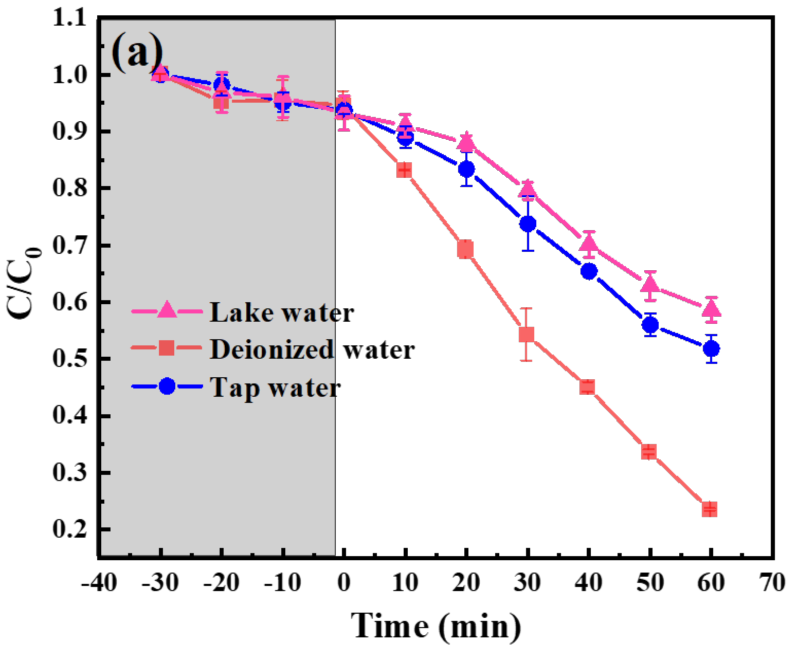

Fig. S4 Actual water quality (tap and lake water) on TC degradation in the photocatalytic degradation of 1CQDs-CNTO30.

Table S1 Energy level parameters: band gap, the position of CB values VB, and flat-band of different catalysts.

catalyst	TiO_2	$g\text{-}C_3N_4$
E_{FB} (*vs. Ag/AgCl*, V)	-1.10	-1.15
E_{FB} (*vs. NHE*, V)	-0.49	-0.54
The distance from VB to the E_f (eV)	2.79	1.80
Valence band (E_{VB}, eV)	2.30	1.26
Band gap (E_g, eV)	3.25	2.67
Conduction band (E_{CB}, eV)	-0.95	-1.41

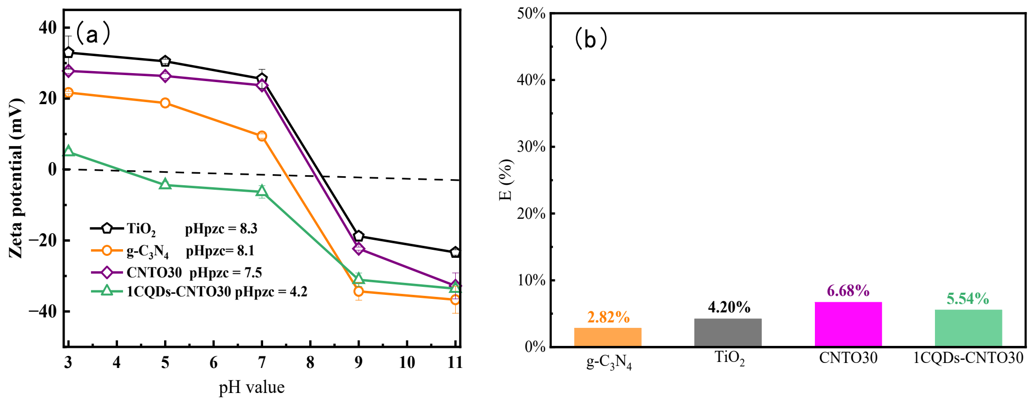

Fig. S5 (a) Surface charge variation as a function of the pH of TiO_2, $g\text{-}C_3N_4$, CNTO30, and 1CQDs-CNTO30, and (b) The adsorption removal efficiency in 30 min

The point of zero charge is a key parameter for predicting the adsorption and photocatalysis behavior to TC. The pH_{PZC} was tested by Zeta potential method [1]. The results present that the pH_{PZC} of TiO_2, $g\text{-}C_3N_4$, CNTO30, and 1CQDs-CNTO30 are 8.3, 8.1, 7.5 and 4.2, respectively. It is found that pH_{PZC} of 1CQDs-CNTO30 significantly reduces to 4.2, meaning there are rich oxygen-containing functional groups introduced

by CQDs. The pH value of the 50 mg L^{-1} TC solution is measured as 6.2. In fact, the dissociation constants (pKa) of TC are 3.3 (pK_{a1}), 7.7 (pK_{a2}), and 9.7 (pK_{a3}). When the pH is 6.2, TC exists in an amphoteric form in the initial stage of the photocatalytic reaction. Therefore, there is a lack of strong electrostatic interaction between the surface charge of all catalysts and TC. Further, the adsorption performance in 30 min of the four materials is also very low (as shown in Fig S5b), which proves that the electrostatic attraction between TC and these four catalysts is very weak.

Reference

[1] S. Kamble, S. Agrawal, S. Cherumukkil, V. Sharma, R.V. Jasra, P. Munshi, Revisiting Zeta Potential, the Key Feature of Interfacial Phenomena, with Applications and Recent Advancements, ChemistrySelect, 7 (2022) e202103084.
